# Non-homologous end joining repair in *Xenopus* egg extract

**DOI:** 10.1038/srep27797

**Published:** 2016-06-21

**Authors:** Songli Zhu, Aimin Peng

**Affiliations:** 1 Department of Oral Biology, College of Dentistry, University of Nebraska Medical Center, Lincoln, NE 68583, USA

## Abstract

Non-homologous end joining (NHEJ) is a major DNA double-strand break (DSB) repair mechanism. We characterized here a series of plasmid-based DSB templates that were repaired in *Xenopus* egg extracts via the canonical, Ku-dependent NHEJ pathway. We showed that the template with compatible ends was efficiently repaired without end processing, in a manner that required the kinase activity of DNA-PKcs but not ATM. Moreover, non-compatible ends with blunt/3′-overhang, blunt/5′-overhang, and 3′-overhang/5′-overhang were predominantly repaired with fill-in and ligation without the removal of end nucleotides. In contrast, 3′-overhang/3′-overhang and 5′-overhang/5′-overhang templates were processed by resection of 3–5 bases and fill-in of 1–4 bases prior to end ligation. Therefore, the NHEJ machinery exhibited a strong preference for precise repair; the presence of neither non-compatible ends nor protruding single strand DNA sufficiently warranted the action of nucleases. ATM was required for the efficient repair of all non-compatible ends including those repaired without end processing by nucleases, suggesting its role beyond phosphorylation and regulation of Artemis. Finally, dephosphorylation of the 5′-overhang/3′-overhang template reduced the efficiency of DNA repair without increasing the risk of end resection, indicating that end protection via prompt end ligation is not the sole mechanism that suppresses the action of nucleases.

DNA double strand breaks (DSBs) are highly toxic lesions that potentially lead to cell death and genomic instability. The cell employs two major evolutionarily-conserved mechanisms, non-homologous end joining (NHEJ) and homologous recombination (HR) to repair DNA DSBs^1^,^2^. HR restores the broken DNA strand using an intact strand as template, and is available in S and G2 phases after replication of chromatin DNA. By comparison, NHEJ directly religates the two broken ends of a DSB, and is accessible in the entire interphase. It has been shown that NHEJ is the major mechanism of DSB repair in mammalian cells. Genetic defects of the NHEJ pathway have been linked to severe combined immunodeficiency (SCID), premature aging, and cancer^3^,^4^,^5^,^6^.

Existing studies of NHEJ have revealed a sophisticated mechanism at the molecular level. Upon the occurrence of DSBs, a Ku heterodimer composed of Ku70 and Ku80 quickly recognizes and binds DSB ends. This initial step of NHEJ is believed to protect the DSB ends and recruit other NHEJ proteins, including DNA-dependent protein kinase catalytic subunit (DNA-PKcs), X-ray cross-complementing protein (XRCC4), XRCC4-like factor (XLF), DNA ligase IV, etc^4^,^5^. DNA-PKcs is activated upon its recruitment to DSBs. In turn, DNA-PKcs autophosphorylation and DNA-PKcs-mediated phosphorylation of other NHEJ proteins regulate the activity and dynamics of repair proteins^4^,^5^,^7^. Ultimately, DNA ligase IV rejoins the broken DSB ends to complete DNA repair. However, processing of DSB ends rendering them ligatable is often required prior to end ligation. The involvement of nuclease Artemis, DNA polymerases μ and λ, and Polynucleotide kinase/phosphatase (PNKP) in end processing has been well established^4^,^5^,^6^.

In principal, the physiological importance of DSB repair is to not only rejoin the DNA ends, but also avoid mutations or loss of genetic information. While HR repair is known to be error-free, the NHEJ mechanism has been long-regarded as being error-prone. For example, loss of end nucleotides may result from end resection as a necessary step to generate ligatable ends during NHEJ. Therefore, knowledge about the detailed mechanism and regulation of end processing will greatly propel our understanding of NHEJ and its involvement in genomic instability and human diseases.

It is well demonstrated that *Xenopus* egg extract responds to DNA damage in a manner very similar to mammalian cells^8^,^9^,^10^,^11^. In the present study, we sought to investigate NHEJ repair in *Xenopus* egg extracts using a plasmid-based assay. In addition to measuring the efficiency of NHEJ, we isolated and analyzed repair products to assess the fidelity of DNA repair and reveal how DSB ends were processed. Our results argued for a surprising level of preference for precise, error-free repair by the NHEJ machinery. The study highlighted a highly variable nature of end processing that is rigorously dependent on the structure of DSB ends. The presence of non-compatible ends or single strand overhangs did not warrant the action of nucleases. Instead, end resection was effectively suppressed even with several types of non-compatible ends. Moreover, our study demonstrated an important role of ATM in the repair of non-compatible ends.

## Results

### DSB repair in *Xenopus* egg extracts via Ku-dependent NHEJ

**Figure 1:**
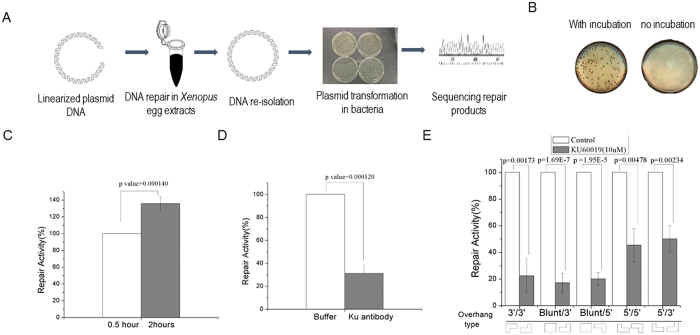
Repair of DSB substrates in *Xenopus* egg extract. (**A**) Schematic diagram of the DSB repair assay. As described in Materials and Methods, linearized plasmid DNA was generated and incubated in *Xenopus* egg extract. Plasmid DNA was then re-isolated from egg extracts, and transformed into bacteria cells. The final repair products were isolated from bacteria colonies and subjected to sequencing analysis. (**B**) As in panel A, incubation of plasmid DNA linearized with Xho1 and Kpn1 in *Xenopus* egg extracts led to formation of colonies after bacteria transformation. As a control, mixing the linearized plasmid with egg extracts without incubation did not yield colony formation. (**C**) As in panel A, the linearized plasmid DNA was incubated in *Xenopus* egg extracts for 0.5 or 2 hr, re-isolated, and transformed into bacteria cells. The repair activity was measured by colony numbers. (**D**) The repair assay was performed in *Xenopus* egg extract that was supplemented with or without Ku70 antibody. The repair activity (in relative to the control extract) was measured by colony numbers. (**E**) The repair assay was performed in *Xenopus* egg extract that was supplemented with or without DNA-PKcs inhibitor (NU7441). The repair activity was measured by colony formation and compared between extracts with or without the inhibitor. Five types of repair templates were used, as in Fig. S2. In panels C–E, a minimum of three experiments were carried out and the results are shown as the mean values and standard deviations. Statistical significance was analyzed using an unpaired 2-tailed Student’s t-test. A p-value < 0.05 was considered statistically significant.

As described in Materials and Methods, we established an *in vitro* system to recapitulate DSB repair in *Xenopus* egg extract, a model that has been widely used to study DNA repair and damage response^8^,^9^,^10^,^11^. Plasmid DNA was linearized by restriction endonucleases (such as Xho1 and Kpn1), and incubated in *Xenopus* egg extracts. The plasmid DNA was then re-isolated from extracts, and transformed into bacteria cells. The repair of the DNA template would result in formation of bacteria colonies, which can be quantified to indicate the efficiency of DNA repair. Moreover, each individual colony contains a single clone of the repair product that can be subjected to sequencing analysis (Fig. 1A). Indeed, the extract possesses biochemical activities to repair DNA DSBs, as judged by the formation of colonies (Fig. 1B). As a control, the same DNA template was mixed with the extract, but the reaction was immediately stopped to prevent DNA repair in the extract. As expected, no colony was formed in the “no incubation” control group, confirming that the formation of colonies was a result of DNA repair in the extract (Fig. 1B). Moreover, a major portion of DNA repair occurred within 30 minutes in extracts (Fig. 1C). The timing of DSB repair in extracts appeared comparable to that in human cells. A previous study showed that NHEJ can be completed in approximately 30 min in human cells, while HR may take several hours^12^. Because our repair substrates did not contain an intact homologous sequence, we reasoned that its repair in extracts was dependent on NHEJ. Consistently, the repair activity decreased significantly when Ku70 antibody was added to the extract (Fig. 1D). Similarly, the repair efficiency was reduced when the extracts were supplemented with specific inhibition of DNA-PKcs (Fig. S1 with NU7026, and Fig. 1E with NU7441). As illustrated in Fig. S2, various endonucleases were used to produce DSB templates with distinct end structures, including blunt/3′-overhang, blunt/5′-overhang, and 3′-overhang/5′-overhang, 3′-overhang/3′-overhang, and 5′-overhang/5′-overhang. Inhibition of the DNA-PKcs kinase activity impaired the repair of all these templates (Fig. 1E).

### The repair of compatible ends requires the kinase activity of DNA-PK but not ATM

**Figure 2:**
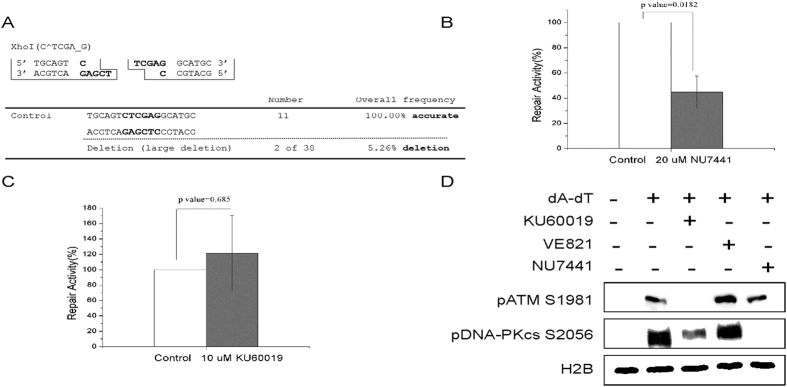
The repair of compatible ends required DNA-PK but not ATM. (**A**) The pMBPII plasmid was digested by XhoI to generate compatible DSB ends. The DNA substrate was incubated in *Xenopus* egg extracts, re-isolated, and transformed into bacteria cells. The final repair products were isolated from bacteria colonies and subjected to sequencing analysis. The repair product with large-fragment deletion (200 bp-2 kb) was determined by agarose gel electrophoresis (data not shown). (**B**) The repair assay was established as in panel A. The extract was supplemented with or without DNA-PKcs inhibitor NU7441. The repair activity (in relative to the control extract) was measured by colony numbers. (**C**) The repair assay was established as in panel A. The extract was supplemented with or without ATM inhibitor KU60019. The repair activity (in relative to the control extract) was measured by colony numbers. (**D**) The specificity of ATM and DNA-PKcs inhibitors were confirmed by immunoblotting. *Xenopus* egg extract were treated with double-stranded oligonucleotides and specific inhibitors as indicated. After incubation for 30 min at room temperature, samples were analyzed using specific antibodies as indicated. In panels B & C, a minimum of three experiments were carried out and the results are shown as the mean values and standard deviations. Statistical significance was analyzed using an unpaired 2-tailed Student’s t-test. A p-value < 0.05 was considered statistically significant.

End processing by nuclease Artemis and other enzymes is an important step of NHEJ. Therefore, an interesting question has been raised with respect to how the NHEJ machinery processes and repairs DSBs that are readily compatible for ligation. This question is important physiologically because DSBs with compatible ends can be frequently induced in the cell by endonucleases, topoisomerases, and other endogenous enzymes. Linearization of plasmid DNA with a single endonuclease produces a DSB repair template with compatible ends. To reveal how DSBs with compatible ends were repaired, we incubated Xho1-digested plasmid DNA in *Xenopus* egg extracts, and isolated multiple repair products for sequencing analysis. Interestingly, all of these products were repaired with direct ligation, as no loss of end nucleotides was observed (Fig. 2A). Therefore, the NHEJ machinery recognizes the compatibility of DSB ends, and effectively suppresses the involvement of end processing nucleases. Consistent with our results, it was previously reported that IsceI-induced DSBs with compatible ends were predominantly repaired by precise ligation in human cells^13^. Two phosphatidylinositol-3-kinase like kinases (PIKK), DNA-PKcs and ATM, have been shown to promote NHEJ in a coordinated and redundant manner^14^,^15^,^16^. We asked if the kinase activity of DNA-PKcs and ATM was required for the precise repair of compatible DSB ends. As shown in Fig. 2B,C, the repair activity decreased with inhibition of DNA-PKcs, but was largely unchanged with ATM inhibition. The specific inhibition of ATM and DNA-PKcs was confirmed by autophosphorylation of ATM and DNA-PKcs (Fig. 2D)

### The repair of non-compatible ends is ATM-dependent

**Figure 3:**
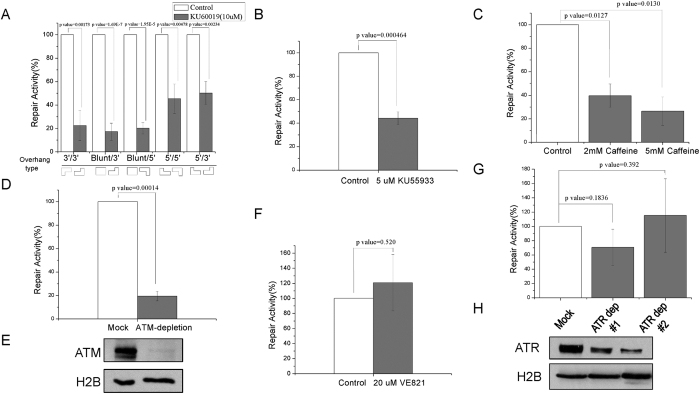
ATM is required for the repair of non-compatible DSB ends. (**A**) *Xenopus* egg extracts were supplemented with or without ATM inhibitor KU60019, and incubated with various types of NHEJ templates as in Fig. S2. The repair activity (in relative to the control extract) was measured by colony numbers. (**B**) *Xenopus* egg extracts were supplemented with or without ATM inhibitor KU55933, and incubated with the 5′/3′ NHEJ template. The repair activity (in relative to the control extract) was measured by colony numbers. (**C**) *Xenopus* egg extracts were supplemented with or without caffeine to inhibit ATM/ATR, and incubated with the 5′/3′ NHEJ template. The repair activity (in relative to the control extract) was measured by colony numbers. (**D,E**) ATM was depleted from*Xenopus* egg extract as described in Materials and Methods. The extract was incubated with the 5′/3′ NHEJ template, and the repair activity (in relative to the control extract) was measured by colony numbers. The efficiency of ATM depletion was confirmed by immunoblotting. (**F**) *Xenopus* egg extracts were supplemented with or without ATR inhibitor VE821, and incubated with the 5′/3′ template. The repair activity (in relative to the control extract) was measured by colony numbers. (**H,G**) ATR was depleted from*Xenopus* egg extract. The extract was incubated with the 5′/3′ NHEJ template, and the repair activity (in relative to the control extract) was measured by colony numbers. The efficiency of ATR depletion was confirmed by immunoblotting. In panels A, B, C, D, F & G, a minimum of three experiments were carried out and the results are shown as the mean values and standard deviations. Statistical significance was analyzed using an unpaired 2-tailed Student’s t-test. A p-value < 0.05 was considered statistically significant.

Five types of non-compatible DSB templates with different end structures were generated as in Fig. S2. Interestingly, the repair of all five templates in *Xenopus* egg extracts was significantly impaired by the inhibition of ATM using KU60019 (Fig. 3A). To rule out the possibility of an off-target effect, we confirmed the requirement of ATM for the repair of the 5′/3′-overhang template using two additional ATM kinase inhibitors, including KU55933 (Figs 3B and S3A) and caffeine (Figs 3C and S3B). Moreover, depletion of ATM from the extract with Mre11-beads disrupted the repair of the the 5′/3′-overhang template (Fig. 3D,E). By comparison, inhibition of ATR (ATM and Rad3-related), another PIKK involved in the DDR, showed no effect on the repair of the 5′/3′-overhang template (Fig. 3F). Consistently, depletion of ATR from the extract with ATRIP-beads also showed no significant effect on the repair (Fig. 3G,H).

### Processing of non-compatible ends is flexible and specific to the structures of DSB ends

**Figure 4:**
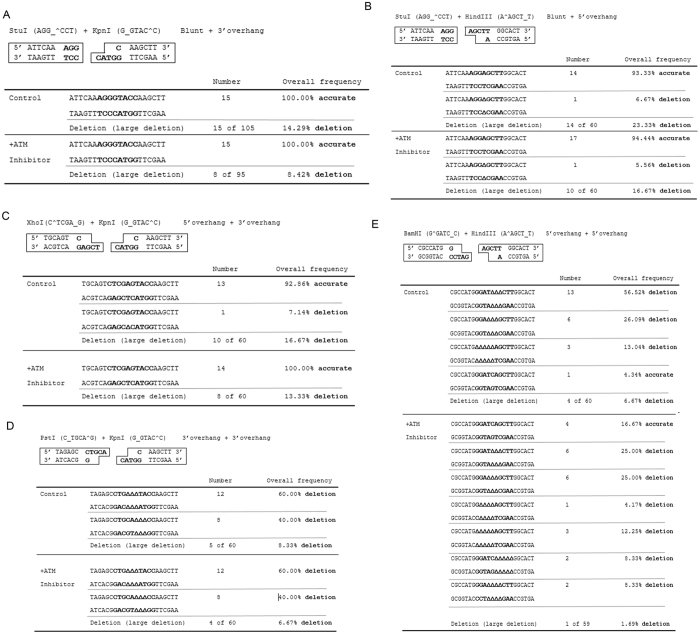
Processing of non-compatible ends is flexible and specific to the structure of DSB ends. Various NHEJ repair templates with non-compatible ends, as in Fig. S2, were incubated in *Xenopus* egg extracts, re-isolated, and transformed into bacteria cells. Final repair products were isolated and subjected to sequencing analysis. The NHEJ templates include: blunt/3′-overhang (**A**), blunt with 5′-overhang (**B**), 3′-overhang/5′-overhang **(C**), 3′-overhang/3′-overhang (**D**), and 5′-overhang/5′-overhang (**E**). The repair assay was performed with or without ATM inhibitor as in Fig. 3B. In each reaction, approximately 15–20 final repair products were sequenced and shown. Nucleotides deleted during DNA repair were indicated by empty triangles. Repair products with large-fragment deletion (200 bp-2 kb) were determined by agarose gel electrophoresis (data not shown).

To reveal detailed insights into how various types of compatible ends were processed during NHEJ, we isolated and sequenced 15–20 repair products for each template. Interestingly, although the existing understanding of NHEJ emphasized the role of end processing nucleases, particularly Artemis, our results argued that the action of nucleases was only involved in the repair of a subset of non-compatible ends. Theoretically, repair templates with a single blunt end, such as blunt/3′-overhang and blunt/5′-overhang, can be processed into ligatable ends via nuclease-dependent removal of the single strand nucleotides. However, our study revealed that in almost all cases, blunt/3′-overhang and blunt/5′-overhang templates were repaired by fill-in of the missing nucleotides (Fig. 4A,B). Moreover, the 3′-overhang/5′-overhang template containing a single strand on either DSB end was also repaired predominantly without end resection (Fig. 4C). In sharp contrast, almost all of the 5′-overhang/5′-overhang and 3′-overhang/3′-overhang templates were repaired with deletion of 3 to 5 nucleotides, confirming the necessary involvement of nucleases in the repair of these substrates (Fig. 4D,E).

As we showed that ATM inhibition decreased the repair efficiency of all non-compatible DSB ends, we asked if ATM inhibition altered the mode of end processing during NHEJ. For blunt/3′-overhang, blunt/5′-overhang, and 3′-overhang/5′-overhang templates that were normally repaired without end resection, ATM inhibition did not lead to loss of end nucleotides in the repair products. In fact, almost all of these substrates were repaired with fill-in and ligation (Fig. 4A–C). By comparison, the 3′-overhang/3′-overhang and 5′-overhang/5′-overhang templates were predominantly repaired with deletion of 3 to 5 nucleotides (Fig. 4D,E). Furthermore, we showed that blunt/3′-overhang, blunt/5′-overhang, and 3′-overhang/5′-overhang templates were largely repaired without end resection with DNA-PK inhibition (Fig. S4). These results emphasized that the end structure represents a dominant determinant in the choice between precise end joining and end resection.

Notably, with all repair templates we observed small portions of repair products that exhibited deletion of large (200 base pair to 2 kb) DNA fragment. This observation is in potential accordance with previous characterization of alternative DSB repair mechanisms, such as alternative NHEJ or single strand annealing^17^,^18^. The risk of large-fragment deletion for compatible ends (5%, Fig. 2A) was significantly lower than that for non-compatible ends (7–23%, Fig. 4A–E). ATM inhibition moderately reduced, but did not block, repair with large-fragment deletion (Fig. 4A–E).

### End dephosphorylation reduced the efficiency of DNA repair without promoting end resection

**Figure 5:**
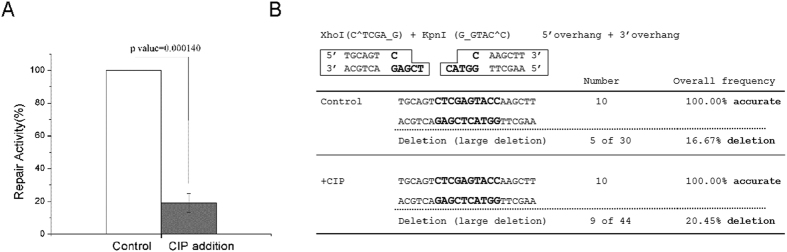
End dephosphorylation reduced the repair efficiency without promoting end resection. (**A**) The 3′-overhang/5′-overhang template was incubated with calf intestinal alkaline phosphatase (CIP), or control buffer, prior to the addition in *Xenopus* egg extracts. The repair activity (in relative to the control extract) was measured by colony numbers. A minimum of three experiments were carried out and the results are shown as the mean values and standard deviations. Statistical significance was analyzed using an unpaired 2-tailed Student’s t-test. A p-value0.05 was considered statistically significant. (**B**) The repair assay with CIP incubation was established as in panel A. Ten final repair products were sequenced and shown. Repair products with large-fragment deletion were determined by agarose gel electrophoresis.

Interestingly, we showed that the 5′-overhang/3′-overhang template containing ssDNA on both ends was repaired without end resection (Fig. 4C). A possible explanation is that both single strand overhangs were immediately ligated by DNA ligase to avoid the recruitment of DNA nucleases. Consistently, it has been proposed that ligases may be recruited to DSBs before nucleases and polymerases, so that compatible ends are ligated to prevent undesired end processing. Because the direct ligation of DSBs requires the presence of a phosphate group at the 5′-DNA terminus, we treated the 5′-overhang/3′-overhang template with calf intestinal alkaline phosphatase (CIP) to remove the 5′ phosphate and delay the ligation of DSB ends in *Xenopus* egg extracts. Interestingly, the repair activity was markedly decreased with CIP-treatment (Fig. 5A). However, the sequence analysis of repair products revealed that all repair products after CIP-treatment were still repaired without loss of end nucleotides (Fig. 5B), indicating that delaying direct end ligation reduced the efficiency of DNA repair, but did not promote nuclease-dependent end processing.

## Discussion

Biochemical delineation of NHEJ in a tractable, *in vitro* system can greatly stimulate the mechanistic investigation of this critical repair process, as well-illustrated in previous studies^19^. Importantly, the evolutionarily conserved nature of the DNA damage response justifies for the use of model systems, including *Xenopus* egg extract that has been utilized as a classic biochemical model. *Xenopus* egg extract is produced by crushing eggs, the simplicity of the process allows an ideal preservation of *in vivo* activities. It has also been well illustrated that *Xenopus* egg extract responds to DNA damage in a manner very similar to mammalian cells^10^,^11^. For example, ATM and ATR-dependent DDR pathway has been extensively investigated in *Xenopus* egg extracts in response to DNA double strand breaks^8^,^9^. In this study, we described a plasmid DNA-based NHEJ assay in *Xenopus* egg extracts. We confirmed that the linearized plasmid DNA is rejoined in *Xenopus* egg extracts via the Ku-dependent NHEJ pathway. In addition to measuring repair efficiency, we isolated individual bacteria colonies and sequenced the repair products to assess the fidelity of DNA repair and reveal how the DSB ends were processed during DNA repair.

While NHEJ is often referred to as an error-prone repair mechanism, emerging evidence argued for an intrinsic precision of this process. A recent study showed that chromosomal DSBs with compatible ends were repaired predominantly by precise end ligation^13^. In this study we showed in *Xenopus* egg extracts that the repair of compatible DNA ends yielded error-free repair products in all cases. The previous study confirmed the role of Ku proteins and DNA ligase IV in previse NHEJ^13^. Here we also demonstrated a requirement of the kinase activity of DNA-PKcs, but not that of ATM, for precise NHEJ. The involvement of DNA-PKcs is intriguing given that DNA-PKcs activity is known to govern neither the end-protection by Ku proteins nor the activity of DNA ligase IV. Interestingly, it has been shown that autophosphorylation of DNA-PKcs was necessary to relieve the physical blockage of end-ligation imposed by DNA-PKcs itself 20,21,22.

We showed in this study that, although the kinase activity of ATM was dispensable for the rejoining of compatible ends, ATM inhibition impaired the repair of all non-compatible ends. By comparison, ATR inhibition did not exhibit an inhibitory effect on NHEJ. Consistent with our finding, previous studies demonstrated a role of ATM in the end resection step of NHEJ via phosphorylation and recruitment of Artemis nuclease^23^. Surprisingly, we reported here that ATM was required for the repair of all five types of non-compatible ends, including those that were repaired without end resection. Therefore, our results pointed to an additional, and uncharacterized role of ATM in end procession. A possible explanation is that the fill-in process of end nucleotides, presumably catalyzed by DNA polymerase μ and λ^24^, is directly or indirectly promoted by ATM. Interestingly, proteomic analysis revealed DNA polymerase λ as a substrate of ATM/ATR^25^. Future efforts shall be directed to characterize ATM-dependent phosphorylation of DNA polymerase λ and other substrates involved in end processing.

**Figure 6:**
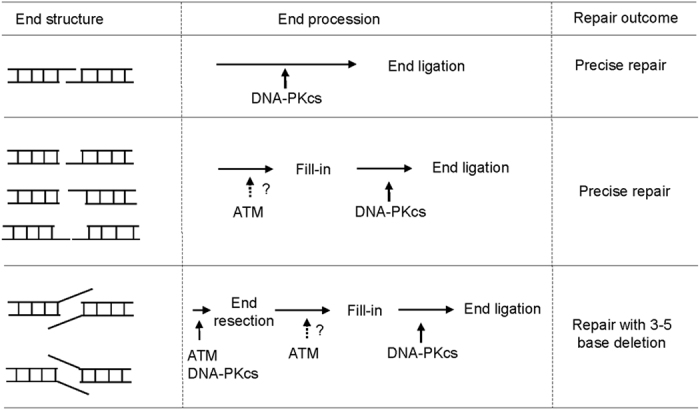
The pattern of NHEJ end processing is specific to the end structure and dependent on ATM and DNA-PKcs. Compatible ends, and non-compatible ends with blunt/3′-overhang, blunt/5′-overhang, and 3′-overhang/5′-overhang were predominantly repaired with fill-in and ligation without resection of end nucleotides. In contrast, 3′-overhang/3′-overhang or 5′-overhang/5′-overhang templates were processed by resection of 3–5 bases and 1–4 base fill-in prior to end ligation. Therefore, the NHEJ machinery exhibited a strong preference for precise repair; the presence of neither non-compatible ends nor protruding single strand DNA sufficiently warranted the action of nucleases. Direct ligation of compatible ends is dependent on the kinase activity of DNA-PKcs but not ATM. ATM was required for the efficient repair of all non-compatible ends, suggesting that ATM-dependent phosphorylation may regulate both end resection and fill-in.

During NHEJ, non-compatible ends have to be first processed to yield compatible ends prior to end ligation. Various enzymes, including DNA nuclease, polymerase, and kinase/phosphatase, have been shown to process DSB ends in NHEJ. Mounting evidence suggested that the recruitment and action of these enzymes in NHEJ can be flexible^4^,^5^,^6^. Our study using different types of repair templates supported the flexibility of end procession, and highlighted the variable nature of end procession in accordance to the specific end structure. We showed that templates with blunt/3′-overhang, blunt/5′-overhang, and 3′-overhang/5′-overhang ends were predominantly repaired with fill-in and ligation, but without resection of end nucleotides. In contrast, 3′-overhang/3′-overhang or 5′-overhang/5′-overhang templates were typically processed via 3–5 base resection and 1–4 base fill-in prior to end ligation. The variable nature of end processing observed in our study is in general agreement with previous studies of NHEJ in human cell lysates, *Xenopus* egg extracts, and reconstitutive assays using purified enzymes^26^,^27^,^28^,^29^,^30^,^31^. The precise involvement of Artemis and other nucleases in the repair of these templates in *Xenopus* egg extracts shall to be examined in future studies. Importantly, our results indicated that the mode of end procession during NHEJ was dictated by the structure of DSB ends. We noted here that the templates with blunt/3′-overhang, blunt/5′-overhang, and 3′-overhang/5′-overhang ends all contains one strand that can be ligated without formation of mismatched or flapping nucleotides on the complementary strand (Fig. 6). Furthermore, we showed that the NHEJ machinery exhibited a strong preference of precise repair devoid of end resection; neither the presence of non-compatible ends, nor that of protruding single strand DNA warranted the action of nucleases. An interesting model is that DNA ligase is recruited to DSB ends prior to other end-processing enzymes, so that compatible ends are directly ligated to avoid undesired end processing. Adding to this idea, even with some non-compatible ends, such as those containing blunt/3′-overhang, blunt/5′-overhang and 3′-overhang/5′-overhang, a single strand may be ligated to avoid end resection. This “ligation *vs* resection competition” model emphasizes the importance of the prompt end ligation for the choice of end processing. However, this model may not fully account for the prevention of end nucleotide removal, as we showed in this study that dephosphorylation of the 5′-overhang/3′-overhang template reduced the efficiency of DNA repair without increasing the risk of end resection. It may be reasoned that the recruitment of NHEJ nuclease also requires certain structural features of DSB ends. In our study, only DSB ends with mismatched and flapping ssDNA were repaired with deletion of 3–5 nucleotides (Fig. 6). Thus, the potential structural determination of DSB ends for the nuclease-dependent end processing remains to be further investigated. Finally, templates used in this study do not fully recapitulate the complex DSBs induced by radiation and oxidation in the cell, and how the NHEJ machinery processes DSBs with complex end-modification will be revealed in future studies.

## Materials and Methods

### Immunoblotting

Sodium dodecyl sulfate-polyacrylamide gel electrophoresis (SDS-PAGE) and immunoblotting were carried out as previously described^32^. Antibodies to ATM, phospho-ATM Ser-1981, phospho-DNA-PKcs Ser-2056 and H2B are obtained from Abcam (Cambridge, MA). Antibodies to ATR and phospho-SMC1 Ser-957 were purchased from Bethyl Laboratories (Montgomery, TX).

### *Xenopus* egg extracts

Cycling extracts were prepared as previously described^32^. Briefly, eggs were rinsed with distilled water then soaked in water for 10 min before being dejellied with 2% cysteine in 1x XB (1 M KCl,10 mM MgCl_2_, 100 mM HEPES pH 7.7, and 500 mM sucrose). Eggs were washed 5 times in 0.2x MMR buffer (100 mM NaCl, 2 mM KCl, 1 mM MgCl_2_, 2 mM CaCl_2_, 0.1 mM EDTA, 10 mM HEPES, and KOH to pH 7.8). Ca^2 + ^ionophore, A23187, was added at 10 ng/ml. When the rotation of animal poles was observed, eggs were washed with 0.2x MMR 10 times and 1x XB 4 times. Eggs were then crushed by centrifugation at 10,000 g at 4 °C. The cytoplasmic layer was transferred to new tubes and added with an energy mix (7.5 mM creatine phosphate, 1 mM ATP, 1 MgCl_2_). The cytoplasmic layer was further separated by centrifugation at 10,000 g for 15 min at 4 °C. Kinase inhibitors used in the study include ATM inhibitors (KU60019, 10 μM; KU55933, 5 μM), ATR inhibitor (VE821, 20 μM), ATM/ATR inhibitor (caffeine 2mM), and DNA-PK inhibitors (NU7441, 20 μM; NU7026, 20 μM).

### DNA repair assay in *Xenopus* egg extracts

Plasmid pMBP-parallel2 (pMBPII) was digested with different restriction enzymes (New England Biolabs, MA) to generate linearized DNA with various end structures. The digested plasmid DNA was purified using a Qiagen DNA purification kit (Valencia, CA), and utilized as substrate of DNA repair. The substrate DNA (20 ng per reaction) was incubated in 20 μl interphase *Xenopus* egg extract at room temperature for DNA repair. The reaction was terminated and stored at −80 °C. As a negative control, the same reaction was established and immediately frozen. The reactions were then thawed, and resuspended in 500 μl of proteinase K Buffer (50 mM Tris HCl to pH 8.0, 150 mM NaCl, 50 mM EDTA to pH 8.0, 0.5% Sarkosyl, 10 ug/ml RNase A and 1 mg/ml proteinase K) at 42 °C for 1 hour. To isolate repair products, reactions were extracted twice with phenol: chloroform, followed by isopropanol precipitation. The purified DNA was transformed into DH5-alpha cells (New England Biolabs, MA), and the colonies were randomly selected and amplified. The plasmid DNA was extracted from DH5-alpha cells using a QIAprep spin miniprep kit (QIAGEN), and analyzed by sequencing.

### ATM and ATR depletion from *Xenopus* egg extracts

For ATM and ATR depletion, pierce glutathione magnetic beads (Thermo Fisher Scientific) were conjugated with GST-Mre11 or ATRIP protein according to the manufacturer’s protocol. Beads were then added to extracts and removed with a magnet after incubation for 30 min. The remaining extract after beads removal was used as for the repair assay, The efficiency of ATM or ATR depletion was assessed by immunoblotting. Mock-depleted extract was prepared similarly with pierce glutathione magnetic beads that were not conjugated with Mre11 or ATRIP protein.

## Additional Information

**How to cite this article**: Zhu, S. and Peng, A. Non-homologous end joining repair in *Xenopus* egg extract. *Sci. Rep.* **6**, 27797; doi: 10.1038/srep27797 (2016).

## Supplementary Material

Supplementary Information
