# Supplementary material for: Non-homologous end joining repair in Xenopus egg extract
Source: Sci Rep. 2016 Jun 21;6:27797. doi: 10.1038/srep27797 (PMC4914968; doi:10.1038/srep27797)
Supplement: Supplementary Information [file srep27797-s1.pdf]

Non-homologous end joining repair in *Xenopus* egg extract

Songli Zhu and Aimin Peng*

Department of Oral Biology, College of Dentistry, University of Nebraska Medical Center, Lincoln, NE 68583, USA.

* To whom reprint requests should be sent: Aimin Peng, Ph.D. Department of Oral Biology, College of Dentistry, University of Nebraska Medical Center, Lincoln, NE 68583. Tel: 402-472-5903, Fax: 402-472-2551. Email: Aimin.Peng@UNMC.edu

Supplemental figure legends

Figure S1. The repair of non-compatible ends was suppressed by DNA-PK inhibition.

Xenopus egg extract was supplemented with or without DNA-PKcs inhibitor NU7026, and incubated with the 5'/3' NHEJ template. The repair activity (in relative to the control extract) was measured by colony numbers. A minimum of three experiments were carried out and the results are shown as the mean values and standard deviations. Statistical significance was analyzed using an unpaired 2-tailed Student's t-test. A p-value <0.05 was considered statistically significant.

Figure S2. Five types of NHEJ templates were generated using different restriction enzymes. Different restriction enzymes were utilized to generate five types of NHEJ templates for the repair assay.

Figure S3. Validation of the ATM and DNA-PK inhibitors.

(A & B) *Xenopus* egg extracts were treated with double-stranded oligonucleotides (dA-dT), and specific inhibitors as indicated. After incubation for 30 min at room temperature, samples were analyzed using specific antibodies as indicated.

Figure S4. The repair of non-compatible ends with DNA-PK inhibition.

Various NHEJ repair templates with non-compatible ends, as in Fig. S2, were incubated in *Xenopus* egg extracts, re-isolated, and transformed into bacteria cells. Final repair products were isolated and subjected to sequencing analysis. The NHEJ templates include: blunt/3'-overhang (A), blunt with 5'-overhang (B), 3'-overhang /5'-overhang (C), 3'-overhang /3'-overhang (D), and 5'-overhang /5'-overhang (E). The repair assay was performed with or without DNA-PK inhibitor (NU7441) as in Fig. 1E. In each reaction, approximately 10 final repair

products were sequenced and shown. Nucleotides deleted during DNA repair were indicated by empty triangles.

Figure S1

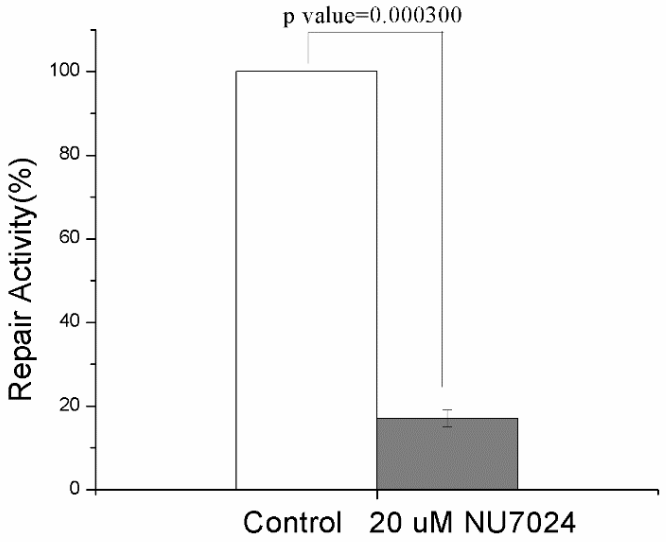
p value=0.000300
Repair Activity(%)
100
80
60
40
20
0
Control
20 uM NU7024

Figure S2

Enzymes	Terminus Configuration	Sequence
StuI / HindIII	Blunt / 5’overhang	AGG AGCTT<br>TCC A
StuI / KpnI	Blunt / 3’overhang	AGG C<br>TCC CATGG
XhoI / KpnI	5’overhang / 3’overhang	C C<br>GAGCT CATGG
PstI / KpnI	3’overhang / 3’overhang	CTGCA C<br>G CATGG
BamHI / HindIII	5’overhang / 5’overhang	G AGCTT<br>CCTAG A

Figure S3

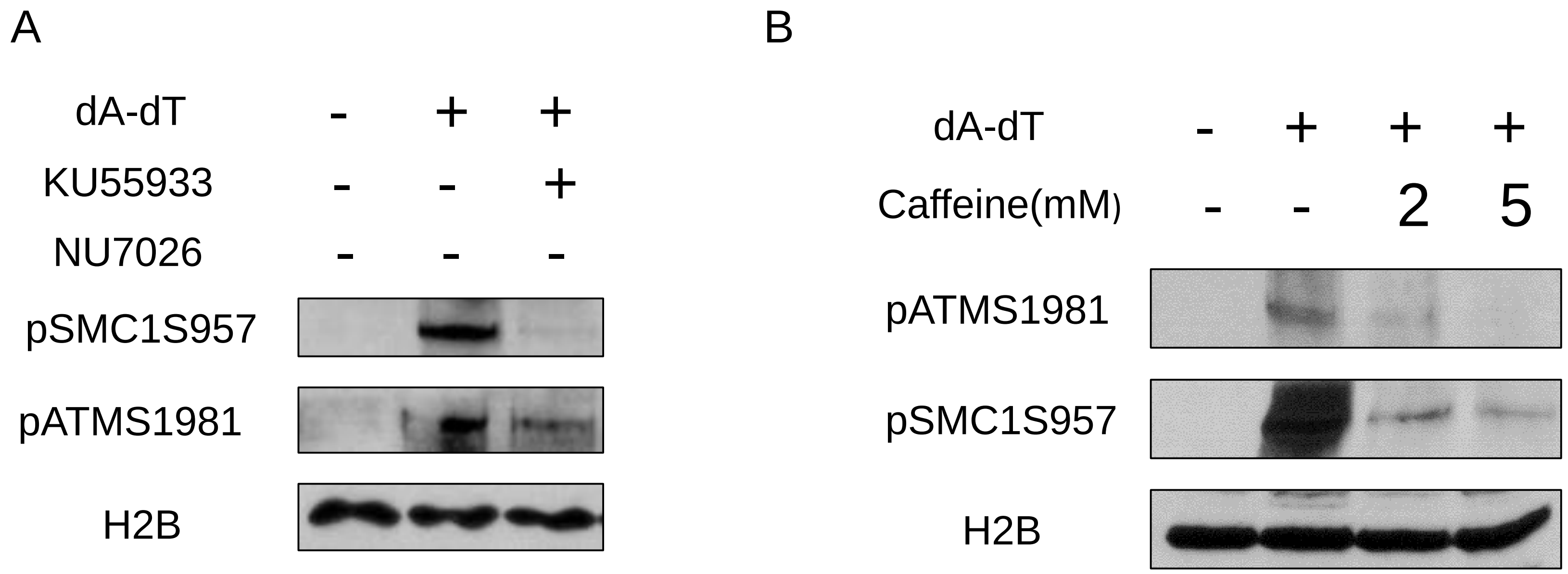
A
dA-dT - + +
KU55933 - - +
NU7026 - - -
pSMC1S957
pATMS1981
H2B
B
dA-dT - + + +
Caffeine(mM) - - 2 5
pATMS1981
pSMC1S957
H2B

Figure S4

A

StuI (AGG_^CCT) + KpnI (G_GTAC^C)   Blunt + 3′overhang

5′ ATTCAA **AGG**   **C** AAGCTT 3′
3′ TAAGTT **TCC**   **CATGG** TTCGAA 5′

		Number	Overall frequency
Control	ATTCAA**AGGGTACC**AAGCTT<br>TAAGTT**TCCCATGG**TTCGAA	10	100.00% **accurate**
+DNA-PK Inhibitor	ATTCAA**AGGGTACC**AAGCTT<br>TAAGTT**TCCCATGG**TTCGAA	10	100.00% **accurate**

B

StuI (AGG_^CCT) + HindIII (A^AGCT_T)   Blunt + 5′overhang

5′ ATTCAA **AGG**   **AGCTT** GGCACT 3′
3′ TAAGTT **TCC**   **A** CCGTGA 5′

		Number	Overall frequency
Control	ATTCAA**AGGAGCTT**GGCACT<br>TAAGTT**TCCTCGAA**CCGTGA	9	90.00% **accurate**
	ATTCAA**AGGΔΔCTT**GGCACT<br>TAAGTT**TCCΔΔGAA**CCGTGA	1	10.00% **deletion**
+DNA-PK Inhibitor	ATTCAA**AGGΔΔΔTT**GGCACT<br>TAAGTT**TCCTCGAA**CCGTGA	8	88.89% **accurate**
	ATTCAA**AGGΔΔΔTT**GGCACT<br>TAAGTT**TCCΔΔΔAA**CCGTGA	1	11.11% **deletion**

C

XhoI(C^TCGA_G) + KpnI (G_GTAC^C)   5′overhang + 3′overhang

5′ TGCAGT **C**   **C** AAGCTT 3′
3′ ACGTCA **GAGCT**   **CATGG** TTCGAA 5′

		Number	Overall frequency
Control	TGCAGT**CTCGAGTACC**AAGCTT<br>ACGTCA**GAGCTCATGG**TTCGAA	8	88.89% **accurate**
	TGCAGT**CTCGΔGTACC**AAGCTT<br>ACGTCA**GAGCΔCATGG**TTCGAA	1	11.11% **deletion**
+DNA-PK Inhibitor	TGCAGT**CTCGAGTACC**AAGCTT<br>ACGTCA**GAGCTCATGG**TTCGAA	11	100.00% **accurate**

D

PstI (C_TGCA^G) + KpnI (G_GTAC^C)   3′overhang + 3′overhang

5′ TAGAGC **CTGCA**   **C** AAGCTT 3′
3′ ATCACG **G**   **CATGG** TTCGAA 5′

		Number	Overall frequency
Control	TAGAGC**CTGΔΔΔTACC**AAGCTT<br>ATCACG**GACΔΔΔATGG**TTCGAA	7	63.64% **deletion**
	TAGAGC**CTGCAΔΔΔCC**AAGCTT<br>ATCACG**GACGTΔΔΔGG**TTCGAA	3	27.27% **deletion**
+DNA-PK Inhibitor	TAGAGC**CTGΔΔΔTACC**AAGCTT<br>ATCACG**GACΔΔΔATGG**TTCGAA	6	66.67% **deletion**
	TAGAGC**CTGCAΔΔΔCC**AAGCTT<br>ATCACG**GACGTΔΔΔGG**TTCGAA	2	22.22% **deletion**
	TAGAGC**CTGCAΔΔΔΔC**AAGCTT<br>ATCACG**GACGTΔΔΔΔG**TTCGAA	1	11.11% **deletion**

E

BamHI (G^GATC_C) + HindIII (A^AGCT_T)   5′overhang + 5′overhang

5′ CGCCATG **G**   **AGCTT** GGCACT 3′
3′ GCGGTAC **CCTAG**   **A** CCGTGA 5′

		Number	Overall frequency
Control	CGCCATG**GGATΔΔΔCTT**GGCACT<br>GCGGTAC**CCTAΔΔΔGAA**CCGTGA	5	50.00% **deletion**
	CGCCATG**GGAΔΔΔGCTT**GGCACT<br>GCGGTAC**CCTΔΔΔCGAA**CCGTGA	3	30.00% **deletion**
	CGCCATG**ΔΔΔΔΔAGCTT**GGCACT<br>GCGGTAC**ΔΔΔΔΔTCGAA**CCGTGA	2	20.00% **deletion**
+DNA-PK Inhibitor	CGCCATG**GGATΔΔΔCTT**GGCACT<br>GCGGTAC**CCTAΔΔΔGAA**CCGTGA	1	10.00% **deletion**
	CGCCATG**GGAΔΔΔGCTT**GGCACT<br>GCGGTAC**CCTΔΔΔCGAA**CCGTGA	1	10.00% **deletion**
	CGCCATG**ΔΔΔΔΔAGCTT**GGCACT<br>GCGGTAC**ΔΔΔΔΔTCGAA**CCGTGA	5	50.00% **deletion**
	CGCCATG**ΔΔΔΔΔΔΔCTT**GGCACT<br>GCGGTAC**ΔΔΔΔΔΔΔGAA**CCGTGA	3	30.00% **deletion**
